# Rigid-Flex PCB Technology with Embedded Fluidic Cavities and Its Application in Electromagnetic Energy Harvesters

**DOI:** 10.3390/mi9060308

**Published:** 2018-06-19

**Authors:** Yi Chiu, Hao-Chiao Hong

**Affiliations:** Department of Electrical and Computer Engineering, National Chiao Tung University, Hsin Chu 30010, Taiwan; hchong@mail.nctu.edu.tw

**Keywords:** printed circuit boards (PCB), rigid-flex, embedded cavity, fluidic, energy harvester, electromagnetic, ferrofluid, magnetic circuit

## Abstract

A technology platform based on commercial printed circuit boards (PCB) technology is developed and presented. It integrates rigid flame retardant (FR)-4 boards, flexible polyimide (PI) structures, and embedded cavities for micro- and meso-scale applications. The cavities or channels can be filled with fluids for microfluidic and lab-on-chip systems. In this study, an electromagnetic energy harvester with enhanced output was designed and implemented in the platform. To enhance harvester output, the embedded cavities were filled with ferrofluid (FF) to improve the overall magnetic circuit design and electromechanical coupling of the device. The fabricated PCB-based harvester had a dimension of 20 mm × 20 mm × 4 mm. Vibration tests of the harvesters were conducted with different magnet sizes and different FF. Test results showed up to a 70% enhancement of output voltage and a 195% enhancement of output power when the cavities were filled with oil-based FF as compared with harvesters without FF. When the cavities were filled with water-based FF, the enhancement of voltage and power increased to 25% and 50%, respectively. The maximum output power delivered to a matched load at a 196-Hz resonance frequency and 1 g_rms_ vibration was estimated to be 2.3 µW, corresponding to an area power density of 0.58 µW/cm^2^ and a volume power density of 1.4 µW/cm^3^, respectively.

## 1. Introduction

Printed circuit boards (PCB) technology is a mature and reliable technology that has been widely adopted in the electronic industry. Standard PCB technology employs both rigid flame retardant (FR)-4 boards and flexible polyimide (PI) films. Multiple rigid and flexible substrates can be laminated and metalized for electrodes and electrical connection. They can also be cut or milled into complex shapes for electronic manufacturing and integration. Therefore, PCB is also a good candidate as a manufacturing technology for electromechanical or mechatronic devices. Another advantage of PCB is that the typical board size of PCB is in the centimeters to tens of centimeters range. Therefore, meso-scale devices and applications can be easily implemented by this technology.

Devices fabricated by PCB technology include various sensors and actuators [1,2]. Vibrational energy harvesters have also been implemented in PCB [3,4,5,6,7,8,9]. In particular, an electromagnetic (EM) energy harvester was demonstrated in Rigid-Flex PCB structures [10]. In further development of this device, embedded cavities were milled in the rigid PCB and filled with ferrofluid (FF) to improve the EM coupling and, thus, enhance output [11]. Detailed device modelling and characterization of the presentation [11] is presented in this paper.

PCB technology has also been suggested as a potential approach for lab-on-PCB and micro total analysis systems (μTAS) applications for its low cost and up-scalability [12]. Various microfluidic devices and systems have been demonstrated [12,13,14]. Most of the PCB-based microfluidic devices used PCB as substrates for sensing electrodes and electrical signal connection. The microfluidic chips were fabricated in other materials and bonded to the PCB substrates for the lab-on-PCB modules. In microfluidic devices based only on standard PCB materials, the channels and fluidic structures have been fabricated in the copper layers [15] or the milled cavities in the PCB laminate [16]. The embedded cavities/channels in PCB presented in this paper complement prior achievements and offer a more versatile tool for implementing complex microfluidic devices.

## 2. Principle and Design

### 2.1. Electromagnetic Energy Harvesters with Embedded Ferrofluid

Vibrational energy harvesters harvest the kinetic energy in ambient vibration sources, such as machinery, buildings, and moving body parts of human and animals. When subjected to an external vibration, the vibrational force causes the shuttle mass in the harvester to move with respect to the rigid device frame. In EM harvesters [3,4,5,10,11,17,18,19], the shuttle mass is a permanent magnet and such displacement causes magnetic flux variation in the surrounding transduction coils. Thus, the vibrational energy is converted to electric energy by the induced electromotive force (EMF) in the coils. In electrostatic harvesters [6,20,21,22], the moving shuttle mass is one of the electrodes of the transduction variable capacitor. The displacement causes charge or potential variation on the moving electrode. Thus, the vibrational energy is converted into the electrostatic energy in the capacitor. In piezoelectric harvesters [23,24,25], the shuttle mass displacement causes deformation of the suspension structures which are made of piezoelectric materials. Thus, the mechanical deformation energy is converted to electricity by the piezoelectric effect. Among these different conversion devices, EM harvesters are attractive because they have simple electrical and mechanical structures, they have low output impedance, and they often use materials that are compatible to modern electronic manufacturing technology.

Harvesters with adequate output power levels can be fabricated by different technologies. Compared with semiconductor-based microfabrication [17,20,21] and traditional precision machining [18,26,27,28], the mature PCB technology has the advantage of low costs and a fast turnaround. Typical minimum line width and feature size in PCB technology is about 100 µm and the available total area of fabricated circuit boards ranges from centimeters to tens of centimeters. Therefore, PCB technology is particularly attractive for manufacturing EM harvesters because the latter often do not have the fine mechanical structures or small electrode gaps seen in other types of harvesters. In addition, PCB can be used as a common substrate to route the coil windings for harvesters and the circuit traces for power processing and control circuits. This enables the development of low-cost and robust integrated wireless sensing modules with minimal components for emerging applications such as the Internet of Things (IoT).

PCB-based energy harvesters have been demonstrated by using both rigid FR-4 boards [3,4,5,7,8] and flexible PI films [6,9]. The rigid boards and flexible films can be laminated in standard processes to form the Rigid-Flex PCB substrates, which have been widely used in consumer products, such as mobile phones and laptop computers which have limited internal space or irregular shapes. The advantage of the Rigid-Flex PCB is that more complicated, versatile, and compact three-dimensional structures can be realized by careful design of the rigid and flexible parts in the device. For example, the Rigid-Flex PCB technology was employed in the folded structure in an electret-based electrostatic harvester where the flexible PI was used as electrical connections and mechanical resonance structures, while the rigid board was used to house the components for power management [6]. In [29], the Rigid-Flex PCB technology was used to fabricate an EM/piezoelectric harvester where the PI layer was used to support the piezoelectric bimorph. In [10], a wide-band EM energy harvester with a more complex rigid-flex structure was demonstrated based on a commercial Rigid-Flex PCB process. This harvester had its transduction coil windings and support mechanical frame designed and fabricated in rigid FR-4 boards, whereas the mass platform and elastic suspension beams were constructed in the flexible PI film. The PI film was sandwiched and laminated between the top and bottom rigid boards to demonstrate the feasibility of Rigid-Flex PCB as both electric substrates and mechanical structure materials.

However, only standard PCB materials, such as copper, fiberglass cloth, and epoxy binder, which were non-magnetic, were used in [10]. Therefore, the magnetic flux from the permanent magnet in the harvester could not be confined and guided efficiently through the transduction coils. To improve the magnetic circuit design and EM conversion efficiency in such devices, a novel rigid-flex-PCB-based EM energy harvester was demonstrated recently [11]. This device had embedded cavities milled in the rigid PCB layers and filled with FF [11]. The high-permeability FF helped guide the magnetic flux of the permanent magnet through the transduction coil and, thus, improve the overall magnetic efficiency of the device. In the following sections, more details of modelling and characterization of the device reported in [11] are presented.

### 2.2. Device Design

**Figure 1:**
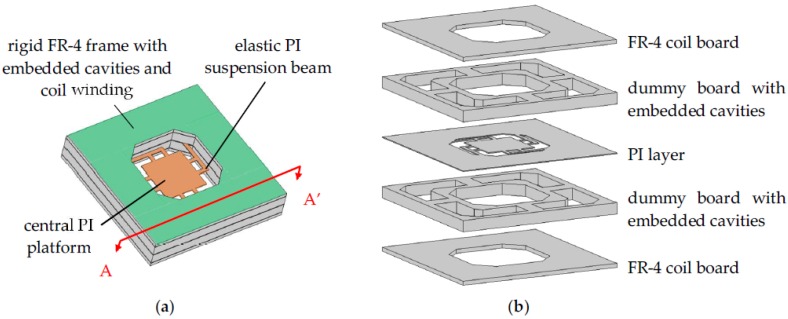
Proposed harvester fabricated based on a commercial Rigid-Flex printed circuit boards (PCB) technology, (**a**) schematic view; (**b**) exploded view.

**Figure 2:**
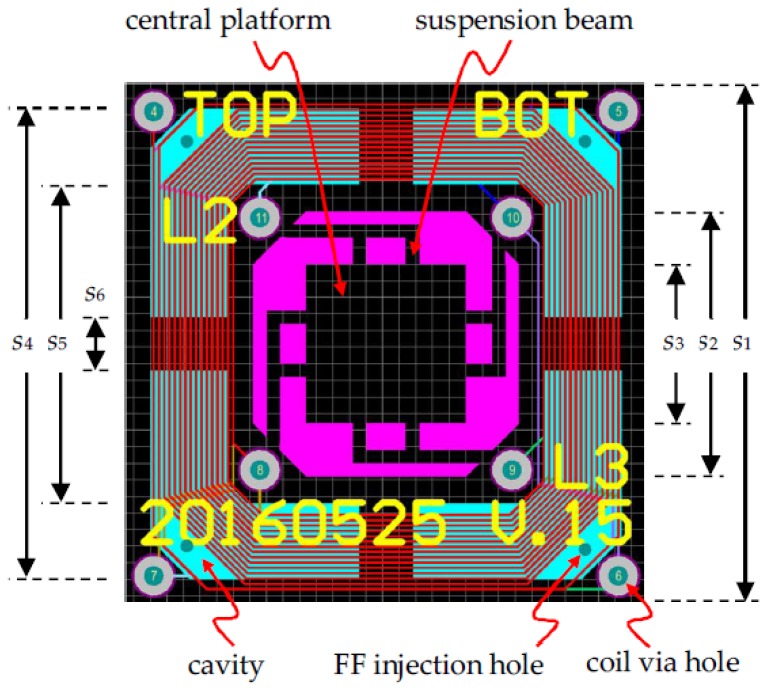
PCB layout of proposed harvester. FF: Ferrofluid.

The schematic view of the proposed device is shown in Figure 1. By using commercial Rigid-Flex PCB technology, the top and bottom transduction coils are routed in FR-4 boards, which are also used as mechanical supports, whereas the elastic suspension beams and the central platform for attached magnets/mass are fabricated in the PI layer. Each of the two sets of coils has three layers of windings connected by via holes (‘via holes’ are holes that connect traces in different layers). The two coils can be connected in series or in parallel in external circuits to increase the output voltage or current, respectively. The central PI layer and the FR-4 coil layers are separated by thick dummy boards which serve two functions: (1) To adjust the positions of the coils to pick up the maximum spatial flux gradient; and (2) to house the embedded cavities for FF, as shown in Figure 1b. After the PCB structure in Figure 1 was fabricated, FF was injected into the embedded cavities in the thick dummy boards. Finally, two NdFeB magnets were attached to the central PI platform and held in place by their strong magnetic force. Figure 2 shows the PCB layout of the proposed harvester. Table 1 summaries the PCB design parameters of the device.

### 2.3. Magnetic Field Calculation

**Figure 3:**
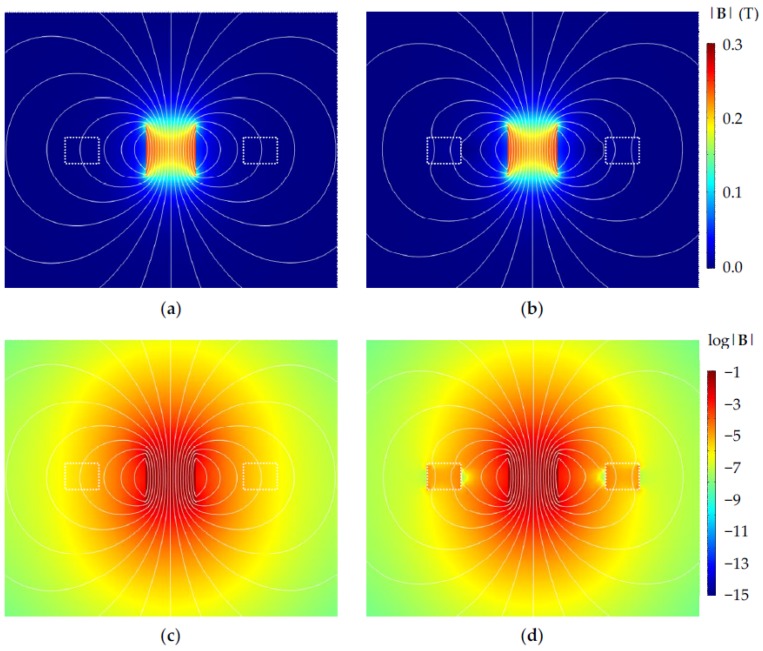
Magnetic flux density calculation showing **B** field magnitude and flux lines. The magnet is in the center and the cavities are outlined in white squares. (**a**,**b**) Cavities without and with ferrofluid (FF), respectively, in linear scale; (**c**,**d**) cavities without and with FF (*µ*_r_ = 18), respectively, in log scale.

In the proposed EM harvester, the magnets move perpendicularly to the planes of the transduction PCB coils. The induced EMF, *V*, can be expressed as:

(1) $$V=-n\frac{d\Phi }{dt}=-n\frac{d\Phi }{dz}\dot{z}$$

where Φ is the total magnetic flux enclosed by the coil, and *z* and $\dot{z}$ are the position and velocity of the permanent magnets in the vertical direction, respectively. Therefore, it is important to achieve maximum spatial flux gradient of Φ to maximize the induced EMF. One of the methods to improve the flux gradient is to increase the total flux enclosed by the coils. This can be accomplished by proper magnetic circuit design of the device. Magnetic circuit design in magnetic actuators and sensors is crucial to enhance the coupling of magnet fields and flux in three-dimensional device structures [1,30,31,32,33]. For example, high-permeability yokes are often used in voice coil motors (VCM) in optical pickup heads in optical disk drives to guide and concentrate magnetic flux and enhance actuation sensitivity [33]. In [19], soft iron was sandwiched between permanent magnets to guide the magnetic flux to enhance the output of an EM energy harvester. In addition to flux guidance, high-permeability materials effectively lower the overall magnetic reluctance in the device and, thus, increase the total magnetic flux. However, the proposed harvesters are designed according to commercial PCB technologies, which do not offer high-permeability magnetic materials. To preserve the manufacturing compatibility with the PCB industry, while keeping the number of components and procedures in the post-PCB assembly steps minimal, a novel yoke design was proposed in the current device by injecting FF into the embedded cavities milled in the dummy boards in the Rigid-Flex PCB stack, as shown in Figure 1. Typically, FF has a relative permeability between 10 and 20. Even though it is not as high as those of ferromagnetic materials, such as Fe or Ni, it is still expected to improve the magnetic design of the proposed harvester. The magnetic field distribution in the device was calculated by using the COMSOL Multiphysics finite-element-method (FEM) software (Ver. 4.3b, Stockholm, Sweden). Figure 3 shows the calculated magnetic flux density, |**B**|, distribution in the PCB-based harvesters without and with injected FF (*µ*_r_ = 18) in the embedded cavities. The remnant flux density of the magnet used in the simulation is the measured value of *B*_r_ = 0.3 T. The induced EMF in the planar coils on the *xy* plane in the device is due to the *z*-component of the magnetic flux density, *B_z_*, only. It can be seen in Figure 3a,b that more magnetic flux lines are attracted to the cavities with FF and they enter the air/FF interface more perpendicularly. These effects enhance the vertical *B_z_* component on the coil plane. Furthermore, Figure 3c,d depicts the flux density in log scale to show that the total B magnitude is, indeed, higher in cavities with FF. 

**Figure 4:**
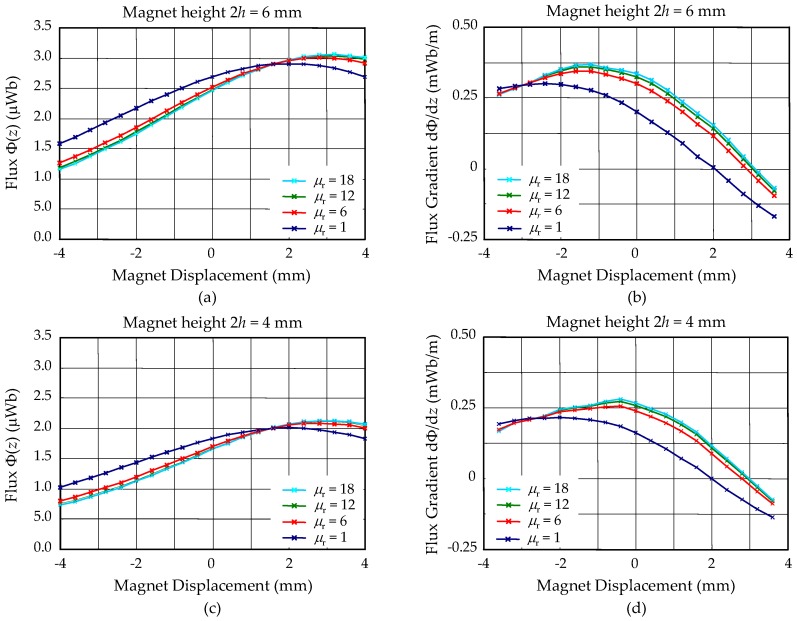
Magnetic flux and flux gradient obtained from finite-element-method (FEM) calculation for various devices configurations. (**a**) Magnetic flux for 2*h* = 6 mm; (**b**) magnetic flux gradient for 2*h* = 6 mm; (**c**) magnetic flux for 2*h* = 4 mm; (**d**) magnetic flux gradient for 2*h* = 4 mm.

After the PCB structure shown in Figure 1 was manufactured, two magnets of a height, *h*, and diameter, ϕ6 mm, were attached to the central PI platform. Therefore, the total height of the magnet mass is 2*h* and the center of the magnet is at *z* = 0. The magnetic flux, Φ(*z*), enclosed by the transduction coil positioned on the top PCB surface (*z* = 2 mm) when the permanent magnet is displaced in the *z* direction is shown in Figure 4. The flux gradient in the *z* direction, dΦ/d*z*, calculated from Φ(*z*), is also shown in Figure 4. Various device configurations were studied, including different magnet heights and FF permeability. The data with *µ*_r_ = 1, corresponding to devices without FF, were used as reference. Several observations can be made from these calculations. 

For devices without FF:

1. The total flux produced by different magnets was approximately proportional to the magnet height 2*h* (Figure 4a,c); and
2. The total flux reached maximum when the magnet centers were aligned with the coil plane at *z* = 2 mm. However, the flux gradient dΦ/d*z* vanishes at this position.

For devices with FF, as compared with devices without FF:

1. The total flux when the magnet was near the rest position (*z* = 0) is lower (Figure 4a,c) whereas the flux gradient at the same position is higher (Figure 4b,d) for devices with FF. The total flux enclosed by the coils had two components, one from inside the magnet, which is positive, and the other from outside the magnet, which is negative. This result indicates that the FF in the cavities contributes more negative flux, relatively, as evidenced by the more perpendicular flux lines shown in Figure 3;
2. The positions for peak flux gradient were shifted toward the rest magnet position by the FF for all different device configurations (Figure 4b,d). Particularly, in Figure 4d, the maximum flux gradient was shifted to approximately align with the rest magnet position at *z* = 0. This indicates that the device with a magnet height of 2*h* = 4 mm, shown in Figure 4d, was operated at the optimal condition. As discussed in the above, the thickness of the dummy board (and, thus, the thickness of the embedded FF cavities and the position of the transduction coil) can be designed according to this calculation to optimize the device operation conditions and maximize harvester output; and
3. The enhancement of the flux and flux gradient was relatively insensitive to the selected range of permeability of the injected FF.

## 3. Device Fabrication

**Figure 5:**
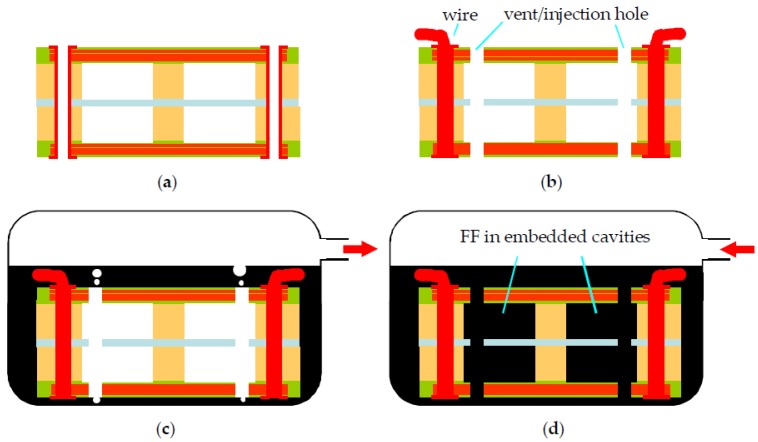
AA’ cross section in Figure 1 showing the FF injection processes. (**a**) Device after standard PCB manufacturing processes; (**b**) Vent/injection hole drilling and wire connection; (**c**) Cavity evacuation; (**d**) FF injection.

The device was fabricated by a commercial PCB manufacturer and only standard materials and processes were used. The PCB fabrication and FF injection processes are shown in Figure 1b and Figure 5. First, three types of PCB substrates were prepared separately by standard commercial PCB processes, as shown in Figure 1b. These boards included: (1) 0.5-mm-thick rigid FR-4 coil boards that have three layers of coil winding without via holes; (2) 1.5-mm-thick dummy boards with milled cavities; and (3) PI films with punched geometry for central platforms and suspension beams. The FR-4 coil boards and the dummy boards were first laminated together so that the central opening for the elastic PI structures could be milled. Subsequently, all rigid boards and PI films were laminated. Via holes for connecting the coil windings in different layers were then drilled and electroplated, as shown in Figure 5a. Next, vent/injection holes for FF injection were drilled through the embedded cavities in the laminated rigid-flex PCB stack (Figure 5b). After connection wires were soldered, the PCB assembly with drilled vent/injection holes was immersed in FF and placed in a desiccator (Figure 5c). The desiccator was then pumped by a mechanical pump so that the air inside the cavities escaped the cavities in bobbles through the vent/injection holes. The immersed PCB was held at low pressure for a few minutes until no new bobbles were observed. Then the desiccator was vented and FF was injected into and filled the cavities automatically by the atmospheric pressure. This pump/vent procedure was repeated a few times to ensure the cavities were fully filled with FF. After the FF was injected, two NdFeB magnets were attached to the central PI platform and the device fabrication and assembly were completed. It is noted that the FF injection was skipped for the reference device without FF.

Two types of FF were used in the study. Table 2 summarizes the properties of the fluid. The total volume of the embedded cavities in the harvesters is estimated to be 0.46 cm^3^. Therefore, the total weight of FF that could be injected into the device was 0.80 g and 0.59 g for the oil-based and water-based FF, respectively. In the FF injection procedure, the total weight of the device was measured before and after the injection. The weight of experimentally injected FF was calculated from these measurements and found to be 0.81 g and 0.56 g for the oil-based and water-based FF, respectively. Therefore, the cavities were nearly fully filled after the injection process.

**Figure 6:**
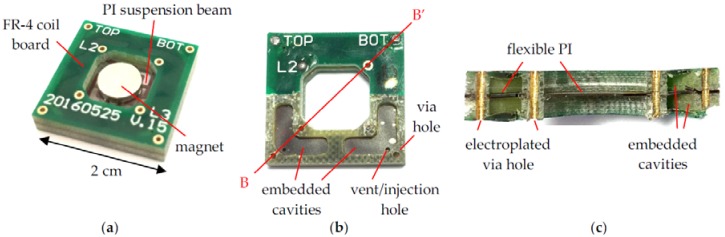
(**a**) Overview of a fabricated harvester with attached magnets; (**b**) top view of a partially delaminated device showing embedded cavities in the dummy board; (**c**) BB’ cross-sectional view of cavities and via holes.

Figure 6a shows a fabricated and assembled harvester with two attached 2-mm-thick NdFeB magnets. As shown in Table 1, the total device dimension in this study is 20 mm × 20 mm × 4 mm, with a central opening of 10 mm × 10 mm in the rigid FR-4 and dummy boards. The measured internal resistance of the coils was close to 70 Ω, with slight variation among devices. Figure 6b shows the embedded cavities and the vent/injection holes drilled in the sandwiched PI film when the top FR-4 coil board was partially delaminated. Figure 6c shows the BB’ cross section of the embedded cavities and the electroplated via holes in the FR-4 coil and dummy boards.

## 4. Vibration Tests and Discussion

**Figure 7:**
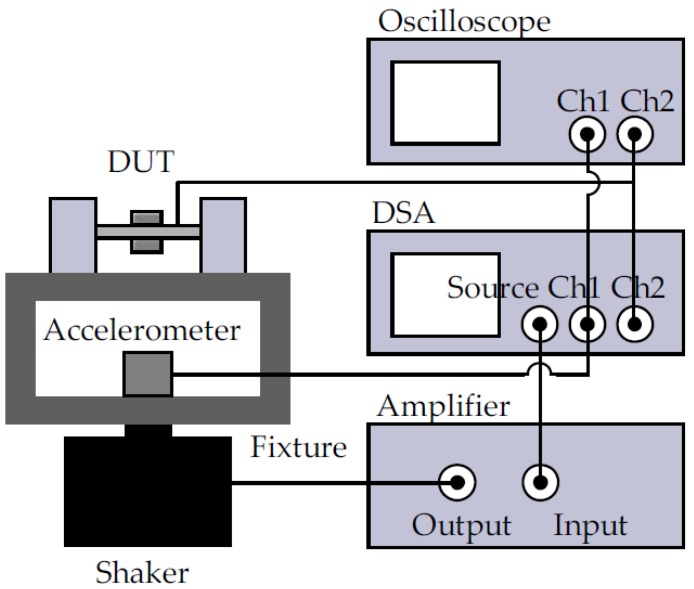
Vibration measurement setup [10].

In the vibration tests, two different cylindrical magnets (ϕ6 mm × 3 mm and ϕ6 mm × 2 mm) were used. Both harvesters with and without FF were tested. The experimental setup for the vibration tests is shown in Figure 7 [10]. The device under test (DUT) and a vibration monitoring accelerometer (352C65, PCB Piezotronics, Depew, NY, USA) were mounted on a shaker (2007E, The Modal Shop, Inc., Cincinnati, OH, USA) co-axially. A dynamic signal analyzer (DSA) (35670A, Keysight Technologies, Santa Rosa, CA, USA) was used to control the test conditions in a closed loop so that the measured vibration level had a constant rms value of 1 g_rms_, with ±0.1 dB tolerance. Vibration tests with both up-sweep and down-sweep of the excitation frequency were conducted and the frequency response of the open-circuited voltage of the harvesters was recorded by the DSA.

**Figure 8:**
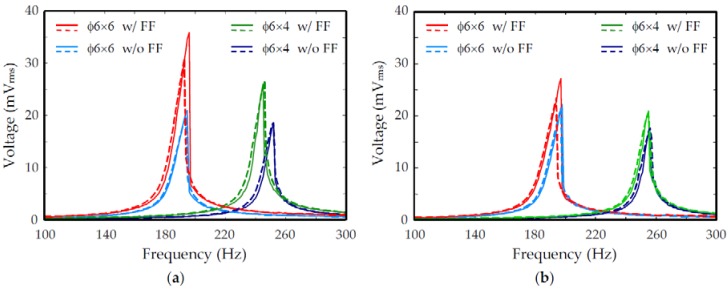
Open-circuited output voltage of proposed harvester with different FF: (**a**) Oil-based FF with *µ*_r_ = 18.6; and (**b**) water-based FF with *µ*_r_ = 12.6. Solid and dash lines were obtained with up and down frequency sweeps, respectively. The magnet size is expressed as ϕ × 2*h* (mm × mm).

Figure 8 shows the measured frequency response of the output voltage, *V*_oc_, for devices filled with different FF. The top and bottom coils were connected in series in these measurements to increase output voltage. The performance characteristics obtained from Figure 8 are summarized in Table 3. For the oil-based FF with *µ*_r_ = 18.6, Figure 8a shows that the ratios of maximum output voltages at resonance with and without FF are 1.7 and 1.4, corresponding to a 70% and 40% increase of voltage for the two types of magnets with heights, 2*h*, of 6 mm and 4 mm, respectively. Therefore, the output power was expected to increase by 195% and 100%, respectively. For the water-based FF with *µ*_r_ = 12.6, Figure 8b shows that the maximum output voltage at resonance was increased by 25% and 20% for the two types of magnets with heights, 2*h*, of 6 mm and 4 mm, respectively. Correspondingly, the output power was expected to increase by 50% and 40%, respectively. Therefore, the enhancement of output voltage and output power by FF in the embedded cavities in the Rigid-Flex-PCB-based EM harvesters was successfully demonstrated. For matched loads, the expected maximum output power, *P*_max_, delivered by a harvester filled with oil-based FF at 1 g_rms_ vibration was 2.3 µW and 1.3 µW for the magnet heights, 2*h*, of 6 mm and 4 mm, respectively. Similarly, the expected maximum output power, *P*_max_, delivered by a harvester filled with water-based FF at 1 g_rms_ vibration was 1.4 µW and 0.8 µW for the magnet heights, 2*h*, of 6 mm and 4 mm, respectively. The maximum area and volume power densities obtained in these experiments were 0.58 µW/cm^2^ and 1.4 µW/cm^3^, respectively, for the harvester with the higher-permeability FF (*µ*_r_ = 18.6) and larger magnet (2*h* = 6 mm). 

### 4.1. Discussion

#### 4.1.1. Normalized Output

From Equation (1), the induced EMF can be written as:

(2) $$V(\omega )=-n\Phi^{\prime }{|}_{0}\omega Z$$

where *V*(*ω*) is the output voltage amplitude, $\Phi^{\prime }{|}_{0}$ is the flux gradient at *z* = 0, as shown in Figure 4b,d, *Z* is the displacement amplitude of the magnet in the harvester, and *ω* is the frequency of the sinusoidal excitation input. It is known that the peak response of a spring-damper-mass second order system, such as a resonant harvester, is:

(3) $$Z(\omega_{0})=\frac{Qm}{k}A=\frac{QA}{\omega_{0}^{2}}$$

where *A* is the excitation acceleration, *Q* is the quality factor of the system, and $\omega_{0}$ is the resonance frequency of the system. From Equations (2) and (3), the maximum output of the harvester is:

(4) $$V_{\max}=V(\omega_{0})=-n\Phi^{\prime }{|}_{0}\omega_{0}Z(\omega_{0})=-nQA\frac{\Phi^{\prime }{|}_{0}}{\omega_{0}}$$

The measured output can be compared with the above theoretical model. The quality factor, *Q*, in Equation (4) is assumed to be 130, obtained from a similar device at low vibration levels [10]. For the harvester with *µ*_r_ = 18 and 2*h* = 6 mm, the flux gradient, $\Phi^{\prime }{|}_{0}$, obtained from Figure 4b is 3.4 × 10^−4^ Wb/m. Since there are three layers of coils in both top and bottom coil boards, the total number of turns is 6*n* = 90. Thus, the output voltage at resonance, *V*_max_, calculated from Equation (4), is 31.4 mV, which is close to the measured value of 35.9 mV. Similarly, the calculated and measured *V*_max_ for the same harvester without FF (*µ*_r_ = 1) are 18.7 mV and 20.9 mV, respectively, which agree with each other well. 

The enhancement of output due to the embedded FF in the above measurements depends on several factors, such as the permeability of the fluid, dimension of magnets, operation frequency, and device variations due to fabrication errors and uncertainties. Therefore, a common figure of merit is presented for comparison of results from various experimental conditions. A normalized output voltage can be defined as:

(5) $$V_{\mathrm{norm}}=\frac{V_{\max}}{\left(Q\Phi^{\prime }{|}_{0}/\omega_{0}\right)}=-nA$$

Since all the experiments were conducted with the same number of turns, *n*, and input acceleration, *A*, in this study, the normalized *V*_norm_ should have similar values for all experimental conditions. The measured resonant frequency, *f*_0_, maximum output voltage, *V*_oc,max_, and the calculated flux gradient, $\Phi^{\prime }{|}_{0}$, obtained from Figure 4 are summarized in Table 3. The normalized voltage, *V*_norm_, calculated by assuming the same quality factor, *Q*, is also shown in Table 3. It can be seen that despite some variation, the calculated normalized voltage agrees with one another relatively well. One possible reason for the variation is that the quality factor may not be the same for different PCB devices, since the elastic resonance structures are mainly constructed in PI films which may not have very consistent mechanical properties. Another possible reason is that the same PCB devices were used both before and after FF injection to reduce possible variation in PCB components. Therefore, the magnets on a harvester without FF had to be removed after vibration tests so that FF could be injected. After the injection, the magnets were re-attached so that the device could be measured with embedded FF. It is possible that the magnets were not re-attached to the same position where they were removed. Therefore, the resonant behavior and flux gradient picked up by the coils may have changed. Finally, Figure 8 shows clear nonlinear behavior in the measured devices. Since nonlinearity is sensitive to details of the device characteristics and experimental conditions, more elaborated nonlinear modeling may be needed for further investigation.

#### 4.1.2. Harvester Effectiveness

Harvester effectiveness, *E_H_*, is a figure of merit proposed in [34] to normalize and compare the performance of resonant harvesters. It is the ratio of measured output power, *P*_out_, to that of a theoretical velocity damped resonant generator (VDRG):

(6) $$E_{H}=\frac{P_{\mathrm{out}}}{Y_{0}Z_{l}\omega^{3}m/2}$$

where $Y_{0}$ and $Z_{l}$ are the amplitudes of the displacement of the vibration source and the displacement of the mass inside the harvester, respectively. Equation (6) can be rewritten as:

(7) $$E_{H}=\frac{P_{\mathrm{out}}}{mA^{2}Q/2\omega }$$

where *A* is the vibration acceleration magnitude and *Q* is the quality factor of the resonant system. Table 4 shows the calculated effectiveness of a few harvesters, including two from the current study and one from our prior publication [10] before the embedded cavity technology was developed. The effectiveness of harvester #3 is about three times of that of harvester #2 due to the embedded FF. However, for harvesters #1 and #2, both of which had similar PCB structures and did not have embedded FF, the harvester in the current study (#2) has a smaller effectiveness than the prior device (#1). The reason is that the prior device was operated with much significant nonlinearity due to the spring hardening effect even at the same vibration level. The nonlinearity shifted the peak power frequency form 185 Hz to 285 Hz [10] and, thus, increased the apparent effectiveness, according to Equation (7). It is noted that the effectiveness defined in Equations (6) and (7) is based on a linear velocity damped resonant generator (VDRG) model. Thus, the application of these equations in strong nonlinear devices, such as the one in [10], should be treated with caution.

Table 4 also includes other devices in the literature [8,35,36] that have similar materials and device configurations. The Rigid-Flex PCB-based harvesters in our studies have a low effectiveness in general. The reason can be found from the coupling strength, *γ,* and the electrodynamic coupling effectiveness, *ε*, defined in [37]:

$$\gamma =\frac{K^{2}/b}{R_{\mathrm{coil}}}$$

where $K=\Phi^{\prime }{|}_{0}$ is the electromechanical coupling coefficient, $b=\omega_{0}m/Q$ is the damping coefficient of the resonant system, and *R*_coil_ is the series resistance of the coil winding, and:

$$\varepsilon =\frac{\gamma }{1+\gamma }$$

For harvester #3 in Table 4, the coil resistance, *R*_coil_, is about 135 Ω and the electrodynamic coupling effectiveness can be calculated as:

$$\varepsilon \approx \gamma \approx 6\times 10^{-4}$$

which is close to the effectiveness value in Table 4. The low effectiveness is mainly caused by the low electromechanical coupling coefficient, *K*, because the transduction coils were placed relatively far away from the magnet in the current planar PCB structure designs. One possible solution to improve the effectiveness of the PCB-based harvesters is to embed the PI suspension springs in a more complex three-dimensional FR-4 PCB structures so that the coils can be placed closer to the magnet.

The low effectiveness is also caused by the simple magnet arrangement in the present study. Even though it is a low-cost and convenient way to assemble the final device, the magnetic flux distribution of such an arrangement is relatively smooth in space and, thus, the flux gradient, $\Phi^{\prime }{|}_{0}$, is small. To enhance the flux gradient, other magnet arrangements will be investigated in future research. For example, instead of two coaxial magnets whose magnetization are in the same direction, as employed in the present study, the two magnets can be assembled so that their magnetization are in opposite directions. Another possible configuration is the side-by-side arrangement of two opposite magnets, as shown in [37]. Much distorted or concentrated magnetic flux lines in these arrangements can be expected to enhance the flux gradient despite higher assembly costs and potential demagnetization effects. 

#### 4.1.3. Effect of High-Permeability Materials on Harvester Characteristics

**Figure 9:**
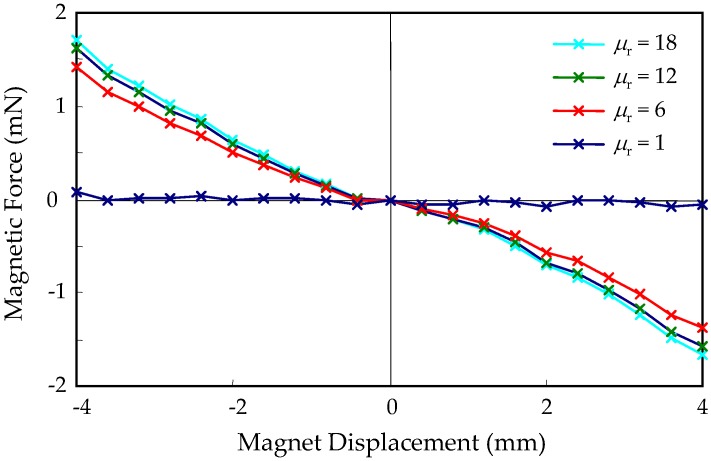
Magnetic force on magnet with 2*h* = 6 mm due to embedded FF.

Embedded FF in the current harvesters not only alters the magnetic field distribution and electromechanical coupling, but introduces a magnetic force interaction with the magnet and changes the dynamics of the harvester. The central rest position of the magnet is a potential minimum due to symmetry. Therefore, the magnetic attraction force between the FF and the magnet is an equivalent spring force. Figure 9 shows the FEM calculation of the restoring magnetic force on a magnet with 2*h* = 6 mm due to the magnetic interaction with FF when the magnet is displaced from its rest position. The equivalent linear spring constant of the force is about 0.4 N/m for the range of permeability of typical FF. Combined with the spring force of the elastic suspension springs, this magnetic spring force can change the total spring constant and, thus, the resonant frequency of the system. The spring constant of the elastic springs can be found from the magnet mass and the resonant frequency of the device to be about 1800 N/m. Therefore, the magnetic spring effect is negligible.

## 5. Conclusions

A platform technology based on commercial Rigid-Flex PCB with embedded cavities was developed and presented. This platform integrates rigid, flexible, and fluidic structures and components and, thus, is suitable for implementation of sensors, actuators, lab-on-PCB, and µTAS. Based on this platform, a novel EM energy harvester was developed. FF was injected into the embedded cavities to enhance the output voltage and output power. Experimental results showed that the cavities were nearly fully filled with the FF by the pump/vent injection process. The measured output voltage was enhanced by 20% to 70% in various device and testing conditions. The corresponding enhancement of the output power was from 40% to 195%. Experiments showed more power could be generated if the embedded FF had a higher permeability and the magnet mass was larger. For 1 g_rms_ vibration levels in the tests, the maximum expected power delivered to a matched load at the resonance frequency of 196 Hz was 2.3 µW, corresponding to an area power density of 0.58 µW/cm^2^ and volume power density of 1.4 µW/cm^3^.

## Figures and Tables

**Table 1:** Design parameters of proposed electromagnetic (EM) harvester. Some parameters are defined in Figure 2.

FR-4 Mechanical Frame Size (mm)		
Outside	*s*_1_	20
Inside	*s*_2_	10
Total thickness	-	4
FF cavity size (mm)		
Outside	*s*_4_	18
Inside	*s*_5_	12.2
Cavity width	(*s*_4_ − *s*_5_)/2	2.9
Gap between cavities	*s*_6_	2
Cavity depth	-	3
PI layer (mm)		
Platform size	*s*_3_	6
Suspension beam width	-	0.5
Coil (mm)		
Line width	-	0.1
Line spacing	-	0.1
Number of turns per layer	*n*	15
FR-4 board thickness	*-*	0.5

**Table 2:** Properties of two types of FF used in experiments.

Type	*µ*_r_	Density (g/cc)	Viscosity (mPa·s)
Oil-based	18.6	1.29	60
Water-based	12.6	1.74	<5

**Table 3:** Performance summary of the proposed harvester under different test conditions.

*µ*_r_	Magnet Size ϕ × 2*h* (mm × mm)	FF	*f*_0_ (Hz)	*V*_oc,max_ (mV_rms_)	*P*_max_ (µW_rms_)	*P*’_max_ (µW_rms_/cm^2^)	$\Phi^{\prime }{|}_{0}$ (mWb/m)	*V*_norm_ (a.u.)
18.6	6 × 6	no	195	20.9	0.78	0.20	0.20	20
		yes	196	35.9	2.30	0.58	0.34	21
	6 × 4	no	252	18.6	0.62	0.15	0.16	29
		yes	246	26.5	1.25	0.31	0.27	26
12.6	6 × 6	no	198	22.2	0.93	0.23	0.20	22
		yes	197	27.1	1.38	0.35	0.33	16
	6 × 4	no	256	17.7	0.59	0.15	0.16	28
		yes	255	20.9	0.82	0.21	0.26	21

Note: *f*_0_: Resonant frequency; *V*_oc,max_: Max open-circuit voltage at resonance; *P*_max_: Max power to matched load at resonance; *P*’_max_: Max area power density to matched load at resonance; $\Phi^{\prime }{|}_{0}$: Flux gradient at zero magnet displacement; *V*_norm_: Normalized open-circuited voltage.

**Table 4:** Harvester effectiveness comparison.

#	Reference	ϕ (mm)	2*h* (mm)	*µ*_r_	*P*_out_ (µW)	*m* (g)	*A*_rms_ (m/s^2^)	*Q*	*f* (Hz)	*E_H_*
1	Prior work [10]	5	4	1	0.34	0.57	9.8	130	285	1.7 × 10^−4^
2	This work	6	6	1	0.78	1.2	9.8	130	195	1.3 × 10^−4^
3	This work	6	6	18	2.30	1.2	9.8	130	196	3.8 × 10^−4^
4	[8]	13	6	--	144	5.36	0.7	23.4	24.4	7.1 × 10^−1^
5	[36]	6	1	--	5	0.2 ^b^	62.3	15 ^c^	390	2.1 × 10^−3^
6	[36]	6	1	--	1	0.2 ^b^	7.6	15 ^c^	370	2.7 × 10^−2^
7	[37]	-- ^a^	-- ^a^	--	0.35	0.16	6.9	33.3	244	4.3 × 10^−3^

^a^ two square magnets with total volume 4 mm × 4 mm × 1 mm; ^b^ estimated from #2; ^c^ estimated from data.

## References

[1] Palasagaram J.N., Ramadoss R. (2006). MEMS-capacitive pressure sensor fabricated using printed-circuit-processing techniques. IEEE Sens. J..

[2] Cetiner B.A., Qian J.Y., Chang H.P., Bachman M., Li G.P., De Flaviis F. (2003). Monolithic integration of RF MEMS switches with a diversity antenna on PCB substrate. IEEE Trans. Microwave Theory Tech..

[3] Podder P., Amann A., Roy S. (2016). Combined effect of bistability and mechanical impact on the performance of a nonlinear electromagnetic vibration energy harvester. IEEE/ASME Trans. Mechatron..

[4] Halim M.A., Rantz R., Zhang Q., Gu L., Yang K., Roundy S. Electromagnetic energy harvesting from swing-arm motion using rotational eccentric mass structure. Proceedings of the 19th International Conference on Solid-State Sensors, Actuators and Microsystems (TRANSDUCERS).

[5] Chen W., Cao Y., Xie J. (2015). Piezoelectric and electromagnetic hybrid energy harvester for powering wireless sensor nodes in smart grid. J. Mech. Sci. Technol..

[6] Chiu Y., Bargayo R.P., Hong H.-C. (2012). Stacked electret energy harvesting system fabricated with folded flexible printed circuit board. Proc. PowerMEMS.

[7] Serre C., Pérez-Rodríguez A., Fondevilla N., Martincic E., Morante J.R., Montserrat J., Esteve J. (2009). Linear and non-linear behavior of mechanical resonators for optimized inertial electromagnetic microgenerators. Microsyst. Technol..

[8] Hapitoglu G., Ürey H. (2010). FR4-based electromagnetic energy harvester for wireless sensor nodes. Smart Mater. Struct..

[9] Chen J., Chen D., Yuan T., Chen X. (2012). A multi-frequency sandwich type electromagnetic vibration energy harvester. Appl. Phys. Lett..

[10] Chiu Y., Hong H.-C., Hsu W.-H. (2016). Wideband vibrational electromagnetic energy harvesters with nonlinear polyimide springs based on rigid-flex printed circuit boards technology. Smart Mater. Struct..

[11] Chiu Y., Hong H.-C. Electromagnetic energy harvester with embedded ferrofluid in PCB technology. Proceedings of the 17th International Conference on Micro and Nanotechnology for Power Generation and Energy Conversion Applications (PowerMEMS).

[12] Moschou D., Tserepi A. (2017). The lab-on-PCB approach: Tackling the μTAS commercial upscaling bottleneck. Lab Chip.

[13] Aracil C., Perdigones F., Moreno J.M., Luque A., Quero J.M. (2015). Portable Lab-on-PCB platform for autonomous micromixing. Microelectron. Eng..

[14] Moschou D., Greathead L., Pantelidis P., Kelleher P., Morgan H., Prodromakis T. (2016). Amperometric IFN-γ immunosensors with commercially fabricated PCB sensing electrodes. Biosens. Bioelectron..

[15] Wego A., Richter S., Pagel L. (2001). Fluidic microsystems based on printed circuit board technology. J. Micromech. Microeng..

[16] Wego A., Glock H.W., Pagel L., Richter S. (2001). Investigations on thermo-pneumatic volume actuators based on PCB technology. Sens. Actuators A.

[17] Liu H., Qian Y., Wang N., Lee C. (2014). An in-plane approximated nonlinear MEMS electromagnetic energy harvester. J. Microelectromech. Syst..

[18] Galchev T., Kim H., Najafi K. (2011). Micro power generator for harvesting low-frequency and nonperiodic vibrations. J. Microelectromech. Syst..

[19] Halim M.A., Cho H., Salauddin M., Park J.Y. (2016). A miniaturized electromagnetic vibration energy harvester using flux-guided magnet stacks for human-body-induced motion. Sens. Actuators A.

[20] Suzuki Y., Miki D., Edamoto M., Honzumi M. (2010). A MEMS electret generator with electrostatic levitation for vibration-driven energy-harvesting applications. J. Micromech. Microeng..

[21] Tvedt L.G.W., Nguyen D.S., Halvorsen E. (2010). Nonlinear behavior of an electrostatic energy harvester under wide- and narrowband excitation. J. Microelectromech. Syst..

[22] Chiu Y., Tseng V.F.-G. (2008). A capacitive vibration-to-electricity energy converter with integrated mechanical switches. J. Micromech. Microeng..

[23] Erturk A., Inman D.J. (2009). An experimentally validated bimorph cantilever model for piezoelectric energy harvesting from base excitations. Smart Mater. Struct..

[24] Shen D., Park J.H., Ajitsaria J., Choe S.Y., Wikle H.C., Kim D.J. (2008). The design, fabrication and evaluation of a MEMS PZT cantilever with an integrated Si proof mass for vibration energy harvesting. J. Micromech. Microeng..

[25] Hajati A., Kim S.G. (2011). Ultra-wide bandwidth piezoelectric energy harvesting. Appl. Phys. Lett..

[26] Zhang Q., Kim E.S. (2014). Vibration energy harvesting based on magnet and coil arrays for watt-level handheld power source. Proc. IEEE.

[27] Khan F., Sassani F., Stoeber B. (2014). Nonlinear behaviour of membrane type electromagnetic energy harvester under harmonic and random vibrations. Microsyst. Technol..

[28] Liu H., Gudla S., Hassani F.A., Heng C.H., Lian Y., Lee C. (2015). Investigation of the nonlinear electromagnetic energy harvesters from hand shaking. IEEE Sens. J..

[29] Wurz M.C., Kleyman G., Twiefel J. (2013). Highly-integrated energy harvesting device for rotational applications utilizing quasi-static piezoelectric and electromagnetic generators. Proc. SPIE.

[30] Ahn C.H., Kim Y.J., Allen M.G. (1993). A planar variable reluctance magnetic micromotor with fully integrated stator and coils. J. Microelectromech. Syst..

[31] Rombach P., Steiger H., Langheinrich W. (1995). Planar coils with ferromagnetic yoke for a micromachined torque sensor. J. Micromech. Microeng..

[32] Kerner C., Magnus W., Golubović D.S., Van Haesendonck C., Moshchalkov V.V. (2004). Micron-sized planar transformer for electromagnetic flux guidance and confinement. Appl. Phys. Lett..

[33] Lee K.-T., Kim C.-J., Park N.-C., Park Y.-P. (2003). Improvement of dynamic characteristics for optical pickup actuator by magnetic circuit. Microsyst. Technol..

[34] Mitcheson P.D., Yeatman E.M., Rao G.K., Holmes A.S., Green T.C. (2008). Energy harvesting from human and machine motion for wireless electronic devices. Proc. IEEE.

[35] Hoffmann D., Kallenbach C., Dobmaier M., Folkmer B., Manoli Y. (2009). Flexible polyimide film technology for vibration energy harvesting. Proc. PowerMEMS.

[36] Mallick D., Amann A., Roy S. (2015). A nonlinear stretching based electromagnetic energy harvester on FR4 for wideband operation. Smart Mater. Struct..

[37] Challa V.R., Cheng S., Arnold D.P. (2012). The role of coupling strength in the performance of electrodynamic vibrational energy harvesters. Smart Mater. Struct..

